# Kinetics of hepatitis B surface antigen and estimated glomerular filtration rate in telbivudine-treated hepatitis B patients with different rescue strategies

**DOI:** 10.1371/journal.pone.0237586

**Published:** 2020-08-12

**Authors:** Hsien-Chung Yu, Kung-Hung Lin, Feng-Woei Tsay, Tzung-Jiun Tsai, Pin-Chieh Wu, Yu-Hsun Chen, Yan-Hua Chen

**Affiliations:** 1 Health Management Center, Kaohsiung Veterans General Hospital, Kaohsiung, Taiwan; 2 Department of Nursing, Meiho University, Pingtung, Taiwan; 3 Division of Gastroenterology and Hepatology, Department of Internal Medicine, Kaohsiung Veterans General Hospital, Kaohsiung, Taiwan; 4 Chung Shan Medical University, Taichung, Taiwan; 5 Institute of Health Care Management, Department of Business Management, National Sun Yat-Sen University, Kaohsiung, Taiwan; 6 Department of Family Medicine, Kaohsiung Veterans General Hospital, Kaohsiung, Taiwan; 7 Division of Colorectal Surgery, Department of Surgery, Kaohsiung Veterans General Hospital, Kaohsiung, Taiwan; East Carolina University Brody School of Medicine, UNITED STATES

## Abstract

This study investigated the kinetics of estimated glomerular filtration rate (eGFR) and quantitative hepatitis B surface antigen (qHBsAg) in telbivudine (LdT)-treated chronic hepatitis B (CHB) patients whose treatment was subsequently adjusted with the adding on adefovir or by switching to tenofovir disoproxil fumarate (TDF) as rescue. Of 295 CHB patients initially treated with LdT, 102 of them who subsequently receiving either adding-on adefovir (group A, n = 58) or switching to TDF (group B, n = 44) for more than 24 months were enrolled. Serial eGFR and qHBsAg levels (3 to 6 monthly) in both LdT monotherapy and rescue therapy periods were analyzed retrospectively. Subsequent decline of qHBsAg especially in rescue therapy period were noted (p<0.001 and p = 0.068 in group A and B). However, patients in group B achieved a significant increase of eGFR (p = 0.010) in LdT monotherapy period but had a significant decline of eGFR (p<0.001) in rescue therapy period. In contrast, patients in group A maintained eGFR levels in both periods. Meanwhile, switch to TDF (hazard ratio: 3.036; 95% confidence interval: 1.040–8.861; p = 0.042) was the sole factor related to the decrease of eGFR>20% from baseline. Both rescue therapies achieved subsequent declines of qHBsAg over time but caused different changes in eGFR. LdT-based rescue therapy maintained eGFR but TDF switching therapy descended eGFR. Therefore, it is essential to monitor patient’s renal function intensively when switching from LdT to TDF as a rescue strategy.

## Introduction

According to international guidelines and the roadmap concept, both the addition of a nucleotide analogue or simply switching to a more potent drug are suggested for chronic hepatitis B (CHB) patients receiving telbivudine (LdT) therapy who exhibit drug resistance or an insufficient response [1–5]. Switching to tenofovir disoproxil fumarate (TDF) is also regarded as the appropriate rescue therapy for patients with LdT-related myopathy or neuropathy [6, 7]. However, renal toxicity is a major concern in patients receiving adefovir (ADV) or TDF therapy [6–11]. In fact, renal toxicity is always a matter of concern when the use of nucleos(t)ide analogues (NAs) occurs over a prolonged period because the clearance of all NAs must occur via the kidneys [12–14]. Nevertheless, improvements in estimated glomerular filtration rate (eGFR) are desirable in CHB patients undergoing LdT therapy irrespective of the state of the given patient’s chronic hepatitis, cirrhosis, or decompensation [15–17]. Some real-world data have also confirmed these findings even though the underlying mechanisms remain unclear [18–21]. However, there is insufficient data in real-world contexts regarding the renal protective effects of LdT therapy for special populations such as drug resistance, side effects from LdT or insufficiency effect by LdT who need their initial treatment with LdT adjusted through the addition of or switching to other drugs for rescue. Furthermore, addressing this lack of data seems important for the purposes of clinical practice for cases in which LdT is chosen as the initial treatment due to the consideration of renal safety because LdT is no longer recommended as a first-line therapy in recent clinical practice guidelines [1–3, 22].

On the other hand, the quantification of hepatitis B surface antigen (qHBsAg) is now increasingly used to determine the treatment response in CHB patients undergoing oral antiviral therapy [23–28]. The goal of oral antiviral therapy for CHB is achieving HBsAg loss [1–3]. An early and significant qHBsAg decline has been found to predict subsequent qHBsAg declines in patients receiving entecavir or LdT therapy [25–27]. However, long-term entecavir or TDF treatment achieved only a slow decline in serum qHBsAg levels in most patients [23, 24]. In LdT-treated CHB patients, a rapid decline of > 1 log IU/mL during the first year was found to be predictive of future HBsAg clearance in patients with HBeAg-positive [25]. Nevertheless, the qHBsAg kinetics during long-term LdT therapy, especially in patients who subsequently receive a rescue therapy other than LdT alone, have not been well investigated in the real world.

Consequently, we conducted this retrospective study of our own LdT-treated cohorts. More specifically, we aimed to investigate the kinetics of eGFR and qHBsAg in CHB patients initially treated with LdT whose treatment was subsequently adjusted to a rescue therapy consisting of LdT with the addition of ADV or treatment with TDF alone in a real-world setting in Taiwan. Subgroup analysis of the impacts of these two rescue therapies on renal function and qHBsAg levels was also conducted in order to clarify which patient groups would be more suitable for which treatment. In addition, we used the Chronic Kidney Disease Epidemiology Collaboration (CKD-EPI) formula [29] for the calculation of eGFR in light of its greater accuracy in patients with normal or slightly impaired renal function, a description which reasonably approximates our patient population in clinical practice.

## Materials and methods

### Patients

We retrospectively evaluated the consecutive CHB patients seen at Kaohsiung Veterans General Hospital in Kaohsiung, Taiwan, from 2008 to 2012 who were treated with LdT (600 mg) as the initial antiviral therapy and enrolled in three studies (VGHKS97-CT9-08, VGHKS98-CT7-06, and VGHKS11-CT5-14). Those patients who subsequently received rescue therapy through either the addition of ADV or by switching to TDF were enrolled for further evaluation. The reasons for the administration of a rescue therapy were as follows: (1) insufficient response (that is, HBV DNA > 60 IU/mL at month six) according to the roadmap concept [5], (2) genotypic resistance to LdT, or (3) the development of LdT-related side effects such as myopathy or neuropathy. The choice of rescue therapy was determined by each patient’s particular circumstances as well as the extent to which the cost of the given therapy would be covered by the national insurance system of Taiwan. However, switching to TDF was first suggested as the rescue therapy for patients experiencing LdT-related side effects, whereas switching to entecavir (1 mg) paid for by the patient himself or herself was suggested as an alternative rescue therapy. This study was approved by the Ethics Committee and the Institutional Review Board of the Kaohsiung Veterans General Hospital (VGHKS97-CT9-08, VGHKS98-CT7-06, and VGHKS11-CT5-14). Written informed consent was obtained from all individual participants included in these studies. All of the methods were performed in accordance with the Declaration of Helsinki and the relevant guidelines.

All of the patients fulfilled the guidelines of the Asian Pacific Association for the Study of Liver [3] at treatment initiation. None of the patients were co-infection with hepatitis C virus or human immunodeficiency virus. Furthermore, any lamivudine-experienced patients with genotypic resistance would not be treated with LdT as the initial therapy.

### Follow-up monitoring

The serologic markers, hematological and biochemical parameters, and HBV DNA levels of each patient were assessed every 3 months during treatment, while qHBsAg levels were checked at baseline, the third month, the sixth month, and every 6 months thereafter. The host, biochemical, and viral factors at baseline and during treatment were also determined. Serum creatinine levels were assessed every 3 months for safety reasons. The eGFR was calculated using the CKD-EPI formula [29]. In patients with acute kidney injury during treatment due to the concomitant usage of nephrotoxic drugs or from other etiologies, the eGFR data from the periods of acute kidney injury would be excluded from the analysis. The dose of LdT, ADV, and/or TDF provided to a patient would be adjusted according to changes in eGFR if indicated [8]. In patients with viral breakthrough (HBV DNA increase > 1 log IU/mL above the nadir), genotypic resistance would be checked. For patients with viral breakthrough with or without genotypic resistance to LdT, rescue therapy with adding-on ADV or switching to TDF would be applied as soon as possible according to the given patient’s choice and the payment of national insurance in Taiwan.

### Biochemistry and laboratory methods

HBV DNA levels were measured using Abbott Real Time HBV assays (Abbott Molecular Inc, Des Plaines, IL, USA) with a lower detection limit of 10 IU/mL. HBV genotype and genotypic resistance were determined by direct DNA sequencing (SeqHepB; Abbott Diagnostics, Lake Forest, IL, USA). The qHBsAg level was measured using Architect QT immunoassays (Abbott Diagnostic, Wiesbaden, Germany). Serum HBsAg, HBeAg, and anti-hepatitis B e antibody (anti-HBe Ab) were measured using radioimmunoassay kits (Ausria II-125; Abbott Laboratories, North Chicago, IL, USA). Hematological and biochemical parameters, including serum creatinine, were measured using automatic analyzers in a central laboratory in our hospital.

### Statistical analyses

All statistical analyses were performed using STATA version 10 (STATA Corp, College Station, TX, USA). Pearsonχ^2^ analysis or Fisher’s exact test was used for the comparison of categorical variables, while continuous variables were compared using the Student’s t test or the Mann-Whitney U test where appropriate. The cumulative responses with time were analyzed using the Kaplan-Meier method and log-rank test. Generalized Estimating Equations (GEE) were used to compare the individual changes in eGFR and qHBsAg over time. Group comparisons were conducted using one-way ANCOVA with or without covariate. Cox regression hazard models were used to estimate the factors related to decrease of more than 20% in the eGFR and decline of more than 0.5 log IU/mL in the qHBsAg. Variables with marginal statistical significance (P < 0.1) in the univariate analysis were subjected to multivariate analysis. A two-tailed *p* value of < 0.05 was considered significant in all tests.

## Results

**Fig 1:**
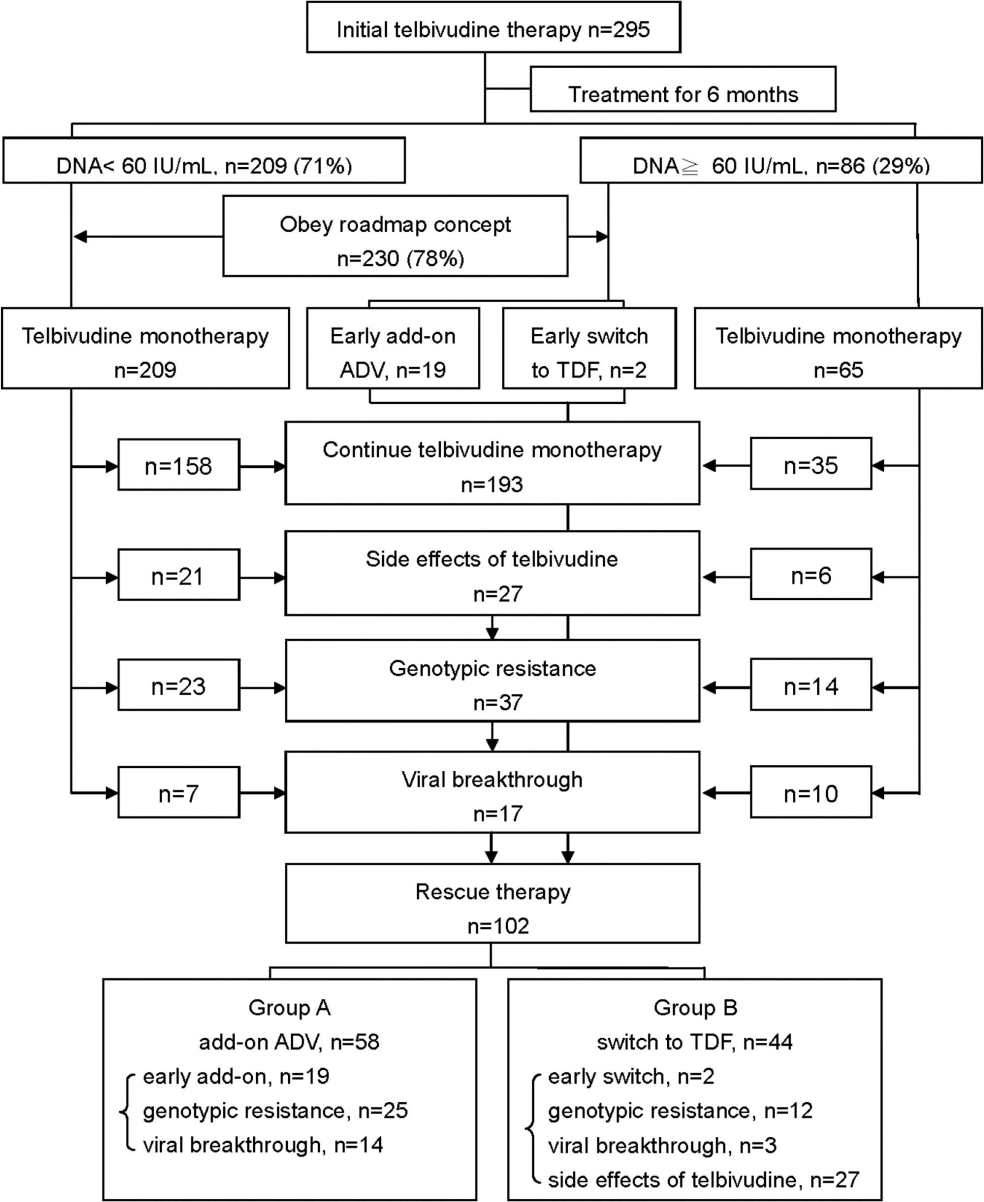
The flow chart of patients who initially received telbivudine therapy and then received a rescue therapy according to the roadmap rule.

A total of 295 patients who received LdT as the initial antiviral therapy from 2008 to 2012 were reviewed. As shown in Fig 1, 209 (71%) patients achieved DNA < 60 IU/mL at month 6 and then continued on LdT monotherapy. For the 86 (29%) patients who did not achieve DNA < 60 IU/mL at month 6, only 21 patients obeyed the roadmap rule and received early rescue therapy beginning at month 6 (with ADV being added for 19 patients and a switch to TDF being applied for 2 patients). The remaining 65 patients requested continued LdT monotherapy and close follow-up. Hence, 230 (78%) of the patients in our real-world cohort obeyed the roadmap rule.

In this LdT-treated cohort, the 102 (34.6%) patients who ultimately received a rescue therapy were enrolled in the present study, and those patients were separated into two groups (Fig 1). The patients in group A (n = 58) received continued LdT with add-on ADV therapy for the reasons of obeying the roadmap rule (n = 19), genotypic resistance to LdT (n = 25), or viral breakthrough (n = 14). The patients in group B (n = 44) underwent a switch from LdT to TDF therapy for the reasons of obeying the roadmap rule (n = 2), genotypic resistance to LdT (n = 12), viral breakthrough (n = 3), or LdT-related myopathy or neuropathy (n = 27). The clinical characteristics of these patients are summarized in Table 1. The patients in group B were older, had a higher rate of cirrhosis, and had higher rates of low HBV DNA levels and low eGFR levels than the patients in group A at baseline. In addition, the S8 Table showed the comparison of the baseline characteristics of the LdT treated patients who early switch to rescue according to roadmap rule (n = 21) or switch to rescue for drug resistance to LdT (n = 54). As shown in this table, the patients in the early switch group had younger age, more HBeAg-positive, more ALT>200 U/L, and more HBV DNA >7 log IU/mL than the patients in the switch for drug resistance group.

**Table 1:** Characteristics of both groups of patients (n = 102) who initially received telbivudine therapy and then received a rescue therapy (at baseline and prior to rescue therapy).

Characteristics	Group A	Group B	P value
	N = 58	N = 44	
Baseline			
Age, years [mean (SD)]	47 (13)	53 (13)	0.014*
Male/ female	42/16	37/7	0.232
Liver cirrhosis, present/ absent	13/45	23/21	0.003*
HBeAg status, positive/ negative	32/26	16/28	0.073
Genotype B/C/unknown	36/17/5	22/17/5	0.820
ALT> 200 U/L, yes/ no	18/40	8/36	0.172
HBV DNA > 7 log IU/mL, yes/ no	27/31	10/34	0.022*
eGFR (CKD-EPI), mL/min/1.73 m^2^	22/35/0/1	11/26/7/0	0.039*
≥90/89-60/59-30/<30			
qHBsAg, IU/mL	21/17/16/4	6/14/18/6	0.052
≥5000/4999-1000/999-100/<100			
Prior to rescue therapy			
LdT monotherapy, months [mean (SD)]	17.5 (15.4)	22.8 (11.9)	0.059
Undetectable DNA, yes/ no	17/41	27/11	0.002*
ALT normalization, yes/ no	33/25	28/16	0.545
HBeAg loss, yes/ no	4/28	1/15	0.652
eGFR (CKD-EPI), ml/min/1.73 m^2^	27/29/1/1	21/19/4/0	0.829
≥90/89-60/59-30/<30			
qHBsAg, IU/mL	10/21/22/5	1/9/31/3	0.004*
≥5000/4999-1000/999-100/<100			

ALT, alanine transaminase; CKD-EPI, the formula of Chronic Kidney Disease Epidemiology Collaboration; eGFR, estimated glomerular filtration rate; HBeAg, hepatitis B e-antigen; LdT, telbivudine; qHBsAg, quantitative hepatitis B surface antigen; SD, standard deviation.

*p< 0.05.

**Fig 2:**
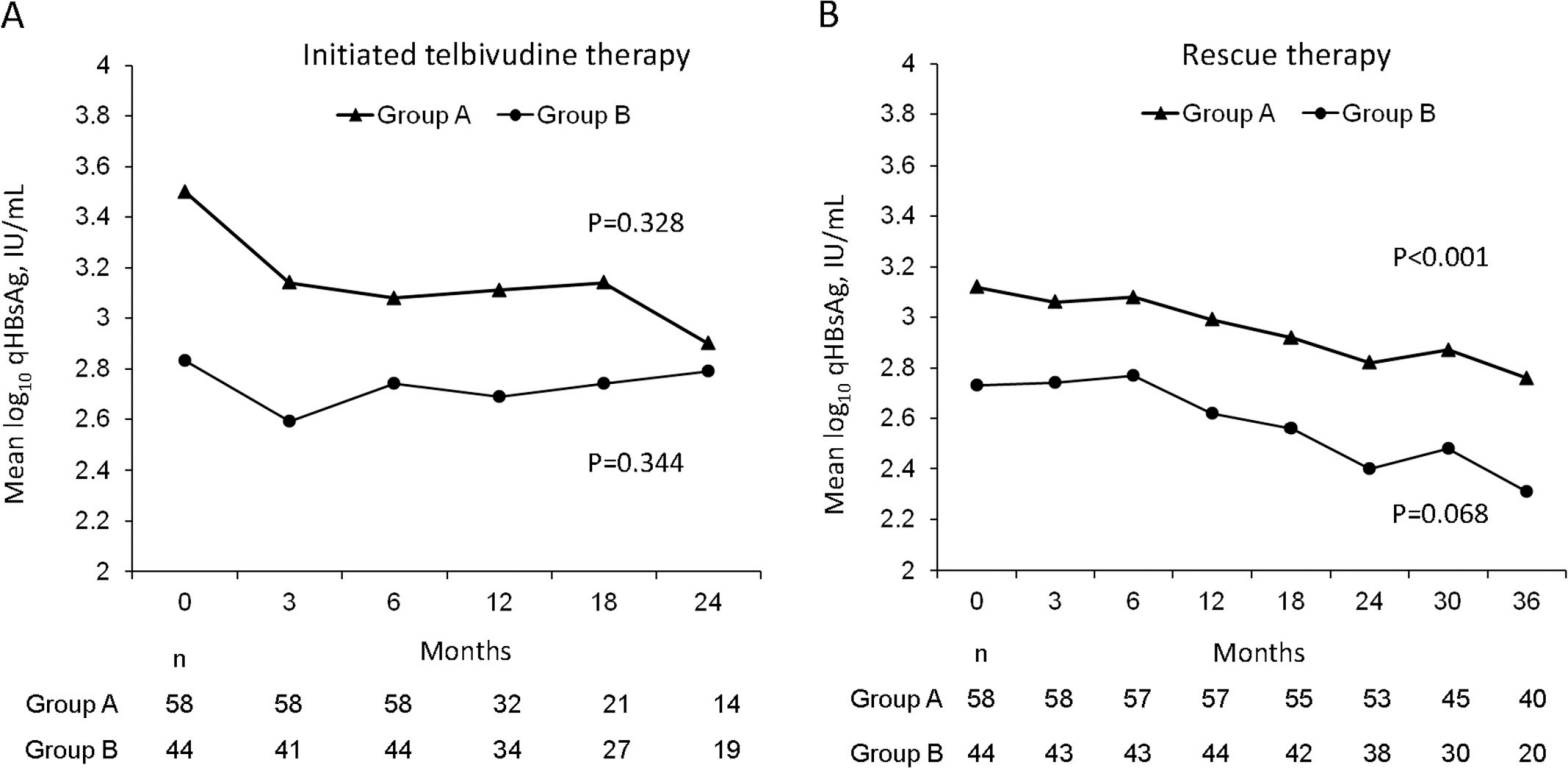
Kinetics of qHBsAg levels in both groups of patients who initially received telbivudine therapy and then received a rescue therapy. The decline of qHBsAg was not significant in either group of patients who initially received telbivudine therapy for 24 months (**A**). Significant and borderline significant declines in qHBsAg, respectively, were noted in the patients in group A (p<0.001) and in the patients in group B (p = 0.068) after receiving rescue therapy for 36 months (**B**). However, the slope of decline was similar in both groups.

**Fig 3:**
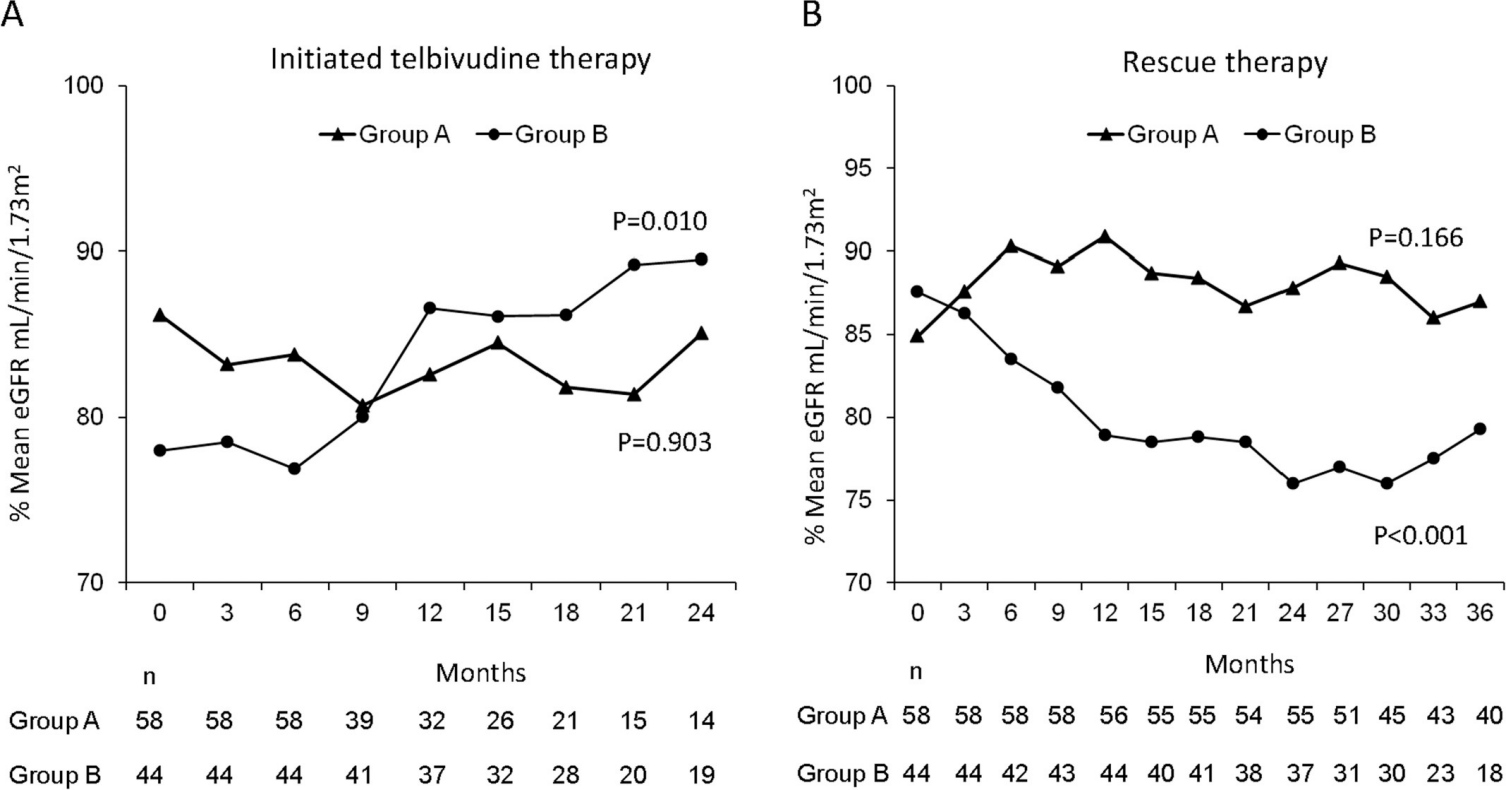
Kinetics of eGFR (by CKD-EPI) levels in both groups of patients who initially received telbivudine therapy and then received a rescue therapy. A significant increase in eGFR was noted in the patients in group B (p = 0.010) but not in the patients in group A (p = 0.903) who initially received telbivudine therapy for 24 months (**A**). On the other hand, a significant decline of eGFR in the patients in group B (p<0.001) and an insignificant change in eGFR in the patients in group A (p = 0.166) were also noted after rescue therapy for 36 months (**B**).

**Fig 4:**
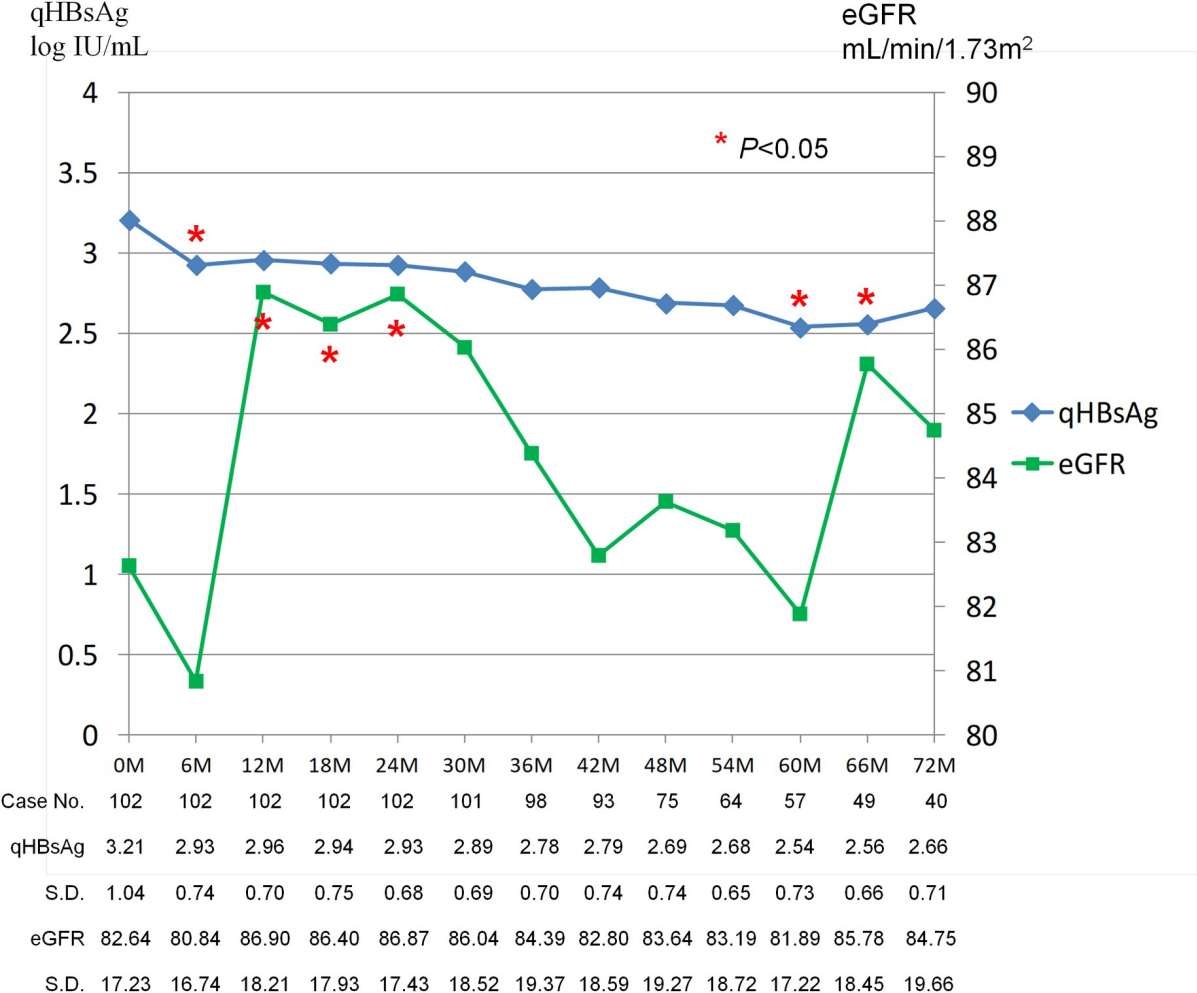
Kinetics of qHBsAg and eGFR (by CKD-EPI) levels in overall patients. Kinetics of qHBsAg and eGFR levels by mean and standard deviation (S.D.) in overall patients from baseline to Month 72. The data that longer than Month 72 are not shown due to small case numbers. The qHBsAg levels decline slowly with the time. The significant increase of eGFR in the first 24 months of treatment were also noted.

The mean durations of the initial LdT monotherapy in group A and group B were 17.5 ± 15.4 and 22.8 ± 11.9 months (p = 0.059) (Table 1), respectively. During these periods, the cumulative rates of alanine aminotransferase (ALT) normalization, DNA negativity, and hepatitis B e antigen (HBeAg) loss across both groups were 60%, 43%, and 12%, respectively. As shown in Table 1, the patients in group B achieved HBV DNA negativity and low qHBsAg levels at higher rates than the patients in group A before rescue therapy. For patients without HBV DNA negativity, the residual viral loads were less than 100,000 IU/mL (range: 87843 IU/ mL to 120 IU/mL), irrespective of whether viral breakthrough occurred or not. However, the decline of qHBsAg was not significant in either group A (p = 0.328) or group B (p = 0.344) within 24 months (Fig 2A). In contrast, a significant increase in eGFR was noted in group B patients (p = 0.010) but not in group A patients (p = 0.903) within the same period (Fig 3A). Subgroup analysis revealed that the benefit of the increases in eGFR were predominant in the group B patients who did not have cirrhosis, did not have genotypic resistance, and were HBeAg-negative (Table 2). In addition, patients with baseline eGFR between 89–60 mL/min had better improvements in renal function before rescue therapy than other patients. About 15 of 61 (24.5%) patients including 5 of 35 patients in group A and 10 of 26 patients in group B were experienced an increase from eGFR between 89–60 mL/min to eGFR > 90 mL/min (Table 1). The kinetics of qHBsAg and eGFR were shown in Fig 4. In general, slowly decline of qHBsAg levels with the time were noted. In contrast, fluctuation of eGFR levels in different time points were noted. However, the significant increase of eGFR in the first 24 months of treatment (most in LdT-based therapy) was achieved.

**Table 2:** Subgroup analysis for the changes of eGFR (by CKD-EPI) levels in both groups of patients who initially received telbivudine therapy and then received a rescue therapy.

Subgroup	Initial telbivudine therapy	P value	Rescue therapy	P value
	Change of eGFR mL/min//1.73 m^2^ (Median; range)		Change of eGFR mL/min//1.73 m^2^ (Median; range)	
HBeAg-positive	-5.3 (-20.4, 18.2) / -6.8 (-12.4, 0.0)	0.698/0.097	2.3 (-18.9, 23.8) /-14.3 (-25.9, -2.2)	0.318/**0.022**
Group A/B				
HBeAg-negative	0.9 (-15.9, 24.1) / 14.6 (-1.3, 41.0)	0.800/**0.001**	3.3 (-33.9, 24.8) / -17.1 (-30.8, -0.7)	0.354/**<0.001**
Group A/B				
Genotype B	0.3 (-20.4, 24.1) / 9.2 (-12.4, 41.0)	0.937/0.078	1.4 (-33.9, 23.8) / -17.3 (-23.8, -5.6)	0.589/**0.002**
Group A/B				
Genotype C	-5.0 (-15.9, 6.0) / 11.9 (-4.4, 35.7)	0.730/0.258	2.5 (-18.9, 24.8) / -14.5 (-30.8, -0.7)	0.472/**0.005**
Group A/B				
Drug resistance	-3.5 (-20.4, 18.2) / 9.4 (-10.5, 35.7)	0.417/0.234	2.3 (-33.9, 24.8) / -9.7 (-18.5, 2.2)	0.514/0.220
Group A/B				
No resistance	5.1 (-13.7, 24.1) / 10.4 (-12.4, 41.0)	0.468/**0.029**	3.0 (-14.1, 23.8)/-17.6 (-30.8, -0.7)	0.162/**< 0.001**
Group A/B				
Cirrhosis	3.2 (-7.7, 16.6) / 6.1(-10.5, 35.7)	0.584/0.166	-2.2 (-33.9, 24.8)/-12.4 (-30.8, -0.7)	0.682/**0.003**
Group A/B				
Non-cirrhosis	0.8 (-20.4, 24.1) / 15.6 (-12.4, 41.0)	0.837/**0.034**	4.2 (-18.9, 23.8) / -20.9 (-27.1, -6.8)	**0.040**/**<0.001**
Group A/B				

CKD-EPI, the formula of Chronic Kidney Disease Epidemiology Collaboration; eGFR, estimated glomerular filtration rate; HBeAg, hepatitis B e-antigen.

The mean durations of rescue therapy in group A and group B were 45.3 ± 17.9 and 33.9 ± 10.7 months (p<0.001), respectively. The cumulative rates of ALT normalization and DNA negativity in group A, group B, and overall were 67%, 82%, 74%, and 72%, 89%, 79%, respectively. The patients in group B achieved higher accumulative rates of DNA negativity than those in group A (p = 0.045), mostly due to their high rates of undetectable HBV DNA before rescue therapy. For the patients without DNA negativity, the residual viral loads were less than 100 IU/mL (from 72 to 12 IU/mL). In addition, the HBeAg loss rates in group A and B, respectively, were 19% and 6% at 2 years and 26% and 13% at 3 years. The patients in group A tended to have higher rates of HBeAg loss than those in group B (p = 0.129).

At 36 months, significant and borderline significant declines in qHBsAg, respectively, were noted in the group A (p<0.001) and in group B (p = 0.068) patients (Fig 2B). Indeed, the slope of decline was similar in both groups. Subgroup analysis revealed similar findings irrespective of the status of HBeAg, genotype, and cirrhosis (Table 3). However, patients with genotypic resistance to LdT were the exception. Among these patients, the patients in group B (p = 0.027) achieved a greater mean decline in qHBsAg than those in group A (p = 0.155). Notably, the patient numbers were relatively small (only 25 patients in group A and 10 patients in group B were treated for up to 36 months). On the other hand, a significant decline of eGFR in group B (p<0.001) and an insignificant change in eGFR in group A (p = 0.166) were also noted at 36 months (Fig 3B). The decline of eGFR in group B predominantly occurred within the first 6–12 months of therapy. Subgroup analysis revealed similar findings irrespective of the status of HBeAg, genotype, and genotypic resistance.

**Table 3:** Subgroup analysis for the changes of qHBsAg levels in both groups of patients who initially received telbivudine therapy and then received a rescue therapy.

Subgroup	Initial telbivudine therapy	P value	Rescue therapy	P value
	Change of qHBsAg IU/mL (Median; range)		Change of qHBsAg IU/mL (Median; range)	
HBeAg-positive	-0.21 (-1.33, 0.47) / 0.26(0.07, 0.24)	0.751/0.145	-0.39 (-2.42, 0.75) / -0.64 (-1.72, 1.11)	**0.007**/0.220
Group A/B				
HBeAg-negative	-0.18 (-2.01, 0.45) / -0.28 (-2.20, 0.89)	0.377/0.197	-0.29 (-1.98, 0.68)/-0.21 (-1.22, 0.86)	**0.047**/0.215
Group A/B				
Genotype B	-0.19 (-2.01, 0.47) / -0.16 (-1.41, 0.89)	0.401/0.458	-0.38 (-2.42, 0.68) / -0.23 (-1.22, 0.86)	**0.012**/0.399
Group A/B				
Genotype C	-0.19 (-0.44, 0.07) / -0.03 (-0.27, 0.09)	0.598/0.755	-0.24 (-0.84, 0.10) / -0.32 (-1.72, 1.11)	**0.004**/0.246
Group A/B				
Drug resistance	-0.34 (-2.01, 0.45) / -0.18 (-0.27, 0.89)	0.237/0.315	-0.25 (-0.87, 0.75) / -0.59 (-1.03. 0.42)	0.155/**0.027**
Group A/B				
No resistance	-0.09 (-0.44, 0.47) / -0.37 (-2.20, 0.67)	0.627/0.155	-0.51 (-2.42, 0.26) / -0.22 (-1.72, 1.11)	**0.002**/0.252
Group A/B				
Cirrhosis	0.25 (-0.04, 0.72) / -0.01 (-1.41, 0.89)	0.151/0.964	-0.46 (-0.91, -0.15)/-0.15(-0.69, 0.86)	**0.001**/0.335
Group A/B				
Non-cirrhosis	-0.21 (-2.01, 0.47) /-0.41 (-2.20, 0.64)	0.317/0.250	-0.32 (-2.42, 0.75) / -047 (-1.72, 1.11)	**0.012**/0.139
Group A/B				

HBeAg, hepatitis B e-antigen; qHBsAg, quantitative hepatitis B surface antigen.

Nevertheless, a significant increase of eGFR was noted among non-cirrhotic patients in particular in group A (p = 0.040) (Table 2).

Since the heterogeneity of baseline characteristics in both groups, multivariate analysis for the significant factors related to both kinetics was further performed (Tables 4 and 5). The baseline HBV DNA > 7 log IU/mL (hazard ratio: 2.208; 95% confidence interval: 1.114–4.377; p = 0.023) and drug resistance (hazard ratio: 0.351; 95% confidence interval: 0.161–0.766; p = 0.009) were two factors related to the decline of qHBsAg > 0.5 log IU/mL from baseline. Notably, there were 1 and 1 patients in group A and B achieved the decline of qHBsAg > 0.5 log IU/mL during the period of LdT monotherapy (p = 0.484). The case numbers were small because of poor viral control and relatively short therapeutic duration in these patients. It was hard to show whether a good decline on LdT monotherapy showing a better decline on rescue therapy in our study. In addition, LdT switch to TDF therapy (hazard ratio: 3.036; 95% confidence interval: 1.040–8.861; p = 0.042) was the sole factor related to the decrease of eGFR > 20% from baseline.

**Table 4:** Factors related to the decline of HBsAg > 0.5 log IU/mL from baseline by univariate and multivariate analysis.

Risk factor	Univariate	P value	Multivariate	P value
	HR(95% CI)		HR(95%CI)	
Age: ≥ 50 years	1.885(0.958–3.708)	0.166		
Sex: male	1.504(0.741–3.052)	0.259		
HBeAg: positive	0.548(0.284–1.056)	0.172		
Cirrhosis	1.258(0.619–2.557)	0.525		
Decompensation	0.728(0.256–2.073)	0.552		
Hepatocellular carcinoma	1.163(0.276–4.900)	0.837		
HBV Genotype: type B	1.178(0.772–1.798)	0.447		
Baseline ALT > 200 U/L	0.980(0.474–2.029)	0.958		
Baseline HBV DNA: > 7 log IU/mL	2.446(1.278–4.680)	0.007	2.208(1.114–4.377)	0.023*
Baseline eGFR < 90 ml/min/1.73m_2_	1.256(0.753–2.095)	0.382		
Diabetes mellitus	0.946(0.365–2.448)	0.908		
Body mass index > 23	0.742(0.389–1.416)	0.365		
Drug resistance	0.361(0.165–0.789)	0.011	0.351(0.161–0.766)	0.009*
LdT switch TDF therapy	0.706(0.367–1.360)	0.299		
Complete virological response at month 6	1.661(0.868–3.178)	0.126		
Baseline HBsAg > 1000 IU/mL	0.513(0.253–1.041)	0.064	0.702(0.323–1.527)	0.372

ALT, alanine transaminase; CI, confidence interval; eGFR, estimated glomerular filtration rate; HBeAg, hepatitis B e-antigen; HBsAg, hepatitis B surface antigen; HR, hazard ratio; LdT, telbivudine; TDF, tenofovir disoproxil fumarate.

*p < 0.05.

**Table 5:** Factors related to the decrease of eGFR (CKD-EPI) > 20% from baseline by univariate and multivariate analysis.

Risk factor	Univariate	P value	Multivariate	P value
	HR(95% CI)		HR(95%CI)	
Age: ≥ 50 years	0.720(0.298–1.738)	0.465		
Sex: male	0.972(0.353–2.679)	0.956		
HBeAg: positive	2.045(0.786–5.325)	0.143		
Cirrhosis	0.249(0.099–0.627)	0.003	0.402(0.150–1.074)	0.069
Decompensation	0.542(0.159–1.851)	0.328		
Hepatocellular carcinoma	0.681(0.157–2.952)	0.608		
HBV Genotype: type B	0.683(0.437–1.169)	0.195		
Baseline ALT > 200 U/L	1.908(0.559–6.513)	0.302		
Baseline HBV DNA: < 7 log IU/mL	1.066(0.424–2.677)	0.892		
Baseline eGFR < 90 ml/min/1.73m_2_	2.042(0.946–4.411)	0.169		
Diabetes mellitus	0.388(0.141–1.069)	0.067	0.574(0.206–1.601)	0.289
Body mass index > 23	0.935(0.387–2.257)	0.881		
Drug resistance	0.853(0.574–1.268)	0.432		
LdT switch TDF therapy	4.452(1.615–12.274)	0.004	3.036(1.040–8.861)	0.042*
Complete virological response at month 6	0.613(0.245–1.538)	0.297		
Baseline HBsAg > 1000 IU/mL	1.390(0.578–3.343)	0.462		

ALT, alanine transaminase; CI, confidence interval; eGFR, estimated glomerular filtration rate; HBeAg, hepatitis B e-antigen; HBsAg, hepatitis B surface antigen; HR, hazard ratio; LdT, telbivudine; TDF, tenofovir disoproxil fumarate.

*p < 0.05.

After rescue therapy, no patient in either group developed multi-drug resistance or viral breakthrough, nor did any patient develop Fanconi syndrome or significant osteoporosis. However, there were 5 (11.4%) patients in group B (the baseline eGFR levels of whom were all between 89–60 mL/min) who had their doses of TDF adjusted because of decreases in eGFR to less than 50 mL/min. Their eGFR levels then fluctuated after adjustment. No patient in group A needed further dose adjustment during rescue therapy. For patients with LdT-related side effects who were switched to TDF therapy, the majority of patients experienced improvements in their symptoms within 6 months after switching, with no patients developing sequelae. In addition, one (1.7%) patient in group A achieved HBsAg seroconversion, while no patients in group B achieved HBsAg loss or seroconversion.

## Discussion

The present study is the first to have investigated the kinetics of qHBsAg levels in CHB patients who initially received LdT therapy and then received a rescue therapy. The results clearly demonstrated a significant decline in qHBsAg with time, irrespective of the type of rescue therapy applied, through the long-term suppression of viral replication. Both the addition of ADV to the initial LdT treatment as well as switching from LdT to TDF achieved good responses in terms of viral control and subsequent declines in qHBsAg in these patients. Moreover, while significant declines in qHBsAg were noted only in the add-on ADV group, not in the switched-to-TDF group, the slope of decline of qHBsAg in both groups was similar. These somewhat discrepant results might be due to different levels of qHBsAg at baseline. In addition, drug resistance was a negative factor related to significant qHBsAg decline because of poor viral control in this study. In contrast, for patients with genotypic resistance to LdT, switching to TDF therapy achieved greater declines in qHBsAg than did add-on ADV therapy. That said, the small number of cases in question could be the reason for that discrepancy. In any case, it is clear that both treatments achieved subsequent declines of qHBsAg with time. These findings were comparable with those of some previous studies in which entecavir or TDF were used for long-term treatment [23, 26, 30]. Hence, it can be concluded that a proper rescue therapy can overcome the risk of drug resistance and achieve similar efficacy in terms of the disease control when LdT is chosen as an initial therapy. On the other hand, the qHBsAg decline were relatively slow irrespective of the antiviral regiments, HBeAg status, HBV genotypes and baseline ALT levels in our study. To my knowledge, most of these patients were hard to treat by LdT monotherapy with poor initial viral control because of drug resistances or insufficiency responses. Only the periods of rescue therapy could achieve HBV DNA negativity in most patients. As we known, the good initial viral control could predict the significant HBsAg decline and subsequent HBsAg loss. In contrast to our patients in the study, the HBsAg-loss cases were minimal because of the short duration of effective rescue antiviral therapy.

However, the two rescue treatments investigated in this study caused differing changes in eGFR. The so-called renal protective effect of the LdT therapy was not hindered by the addition of ADV for rescue in most of the patients in the present study. Moreover, a significant increase in eGFR was demonstrated in non-cirrhotic patients who received the LdT-based rescue therapy. Relatedly, a previous study found that in the case of ADV-based combination therapy, adding on LdT was reported to result in better renal protection than adding on lamivudine or entecavir [31]. In other studies in which the strategy of adding on ADV or TDF was applied, combining either drug with LdT could still result in improved eGFR [32, 33]. Our results were comparable with the results of those previous studies.

In contrast, while the patients in the switched-to-TDF group achieved higher rates of DNA negativity than those in the add-on ADV group in the present study, they experienced significant declines in eGFR within 36 months. The reasons for such declines were complex. Firstly, a randomized controlled trial in Chinese patients reported TDF at licensed dose may had slightly more pronounced reduction in creatinine clearance the ADF [34]. In addition, most of the patients in group B achieved increases in eGFR through LdT monotherapy before switching to TDF. So, the withdrawal of LdT should be one of the reasons for the subsequent eGFR declines in that group because of the removal of the renal protective effect of LdT. However, according to one recent study, the renal protective effect of LdT could persist for at least one year in 48.8% of patients who received LdT therapy for 3 years and then had it withdrawn for one year [35]. Hence, the declines in eGFR seen in the patients in this study could not be explained only by the withdrawal of LdT. Rather, the potential nephrotoxic effect of TDF should also be a reason for it. The decline in eGFR after switching to TDF for rescue has been reported to be significant in the initial 6–12 months in some previous studies [11, 36]. Our results were comparable with the results of those studies. Fortunately, adjustments to doses of TDF or ADV usually decrease the renal toxicity [11, 37]. Moreover, according to the literature, only a minimal number of patients experience Fanconi syndrome and require the withdrawal of TDF [38]. In studies from 2016, meanwhile, tenofovir alafenamide, a newer revision of TDF, was reported to have similar treatments effects to TDF without causing significant bone and renal toxicity [39, 40]. As such, it may serve as an alternative drug of choice for rescue in this kind of patients in the future. However, further study is needed.

Mallet et al. reported that patients who were born in HBV endemic areas and had high initial HBV DNA levels (more than 5 log IU/mL) were more likely to have increased eGFR due to treatment with nucleos(t)ide analogues other than LdT [13]. However, other studies have found the renal protective effects of LdT therapy via the improvement of eGFR to be unique, durable, and unrelated to baseline HBV DNA levels [15–17]. In the present study, high HBV DNA level was a significant factor related to the decline of qHBsAg for more than 0.5 log IU/mL from baseline but not a factor related to the change of eGFR. In addition, the renal protective effects of LdT were particularly notable in non-cirrhotic patients in this study. The cirrhotic patients, meanwhile, seemed to receive only a minimal renal benefit from LdT, although this finding may have been mostly due to the small number of such patients (there were only 13 cirrhotic patients in group A) in this study. In fact, the renal protective effects of LdT in cirrhotic patients have previously been documented in a real-world setting [18].

There were some limitations to the present study. First, we had no cases of initial treatment with LdT with the subsequent addition of TDF that we could consider for comparison. In Taiwan, the national insurance system has paid for add-on ADV therapy since before 2015 and for the switch from LdT to TDF therapy since 2015 for rescue in these kind of patients. Indeed, LdT is used as one of the first line therapies in Taiwan. Nevertheless, when the APASL guideline disclosed in 2012, LdT had been a non-preferred option for the first line therapy in Taiwan. However, for the patients who under LdT-based therapy before, switching therapy or not without conclusion. Our study may provide clinical evidences only for these kind of patients but not for other settings. In addition, this study reflects a real-world scenario and demonstrates the renal protective effect in terms of eGFR of LdT therapy administered under the conditions of routine clinical practice. Second, because the present study was retrospective in nature, we did not check the patients for the possibility of HBV-related renal diseases at baseline and the patients with acute renal injury. There thus might have been some bias because the treatment of hepatitis B with oral antiviral agents can improve renal function in patients with underlying HBV-related renal disease [41]. Finally, some comorbidities or special conditions, such as diabetes, hypertension, pregnant women, and the use of nephrotoxic medications were not well evaluated. We had insufficient data for determining in detail the impact of these factors on the baseline renal statuses of the investigated patients. Nevertheless, only 8 patients (7.8%) had eGFR less than 60 mL/min at baseline in our study. As such, it appeared that the influence of comorbidity on renal function, if any, was not so substantial among these patients. However, further studies are needed to clarify this issue.

In conclusion, both rescue therapies achieved subsequent declines in qHBsAg with time but caused different changes in eGFR. The switch to TDF therapy yielded significant declines in eGFR because of the potential renal toxicity of TDF and the withdrawal of LdT. In contrast, the renal protective effect of the LdT therapy was not hindered by the addition of ADV for rescue. As such, it is essential to monitor patient’s renal function intensively when switching from LdT to TDF is used as a rescue strategy.

## Supporting information

S1 Table Summary of demographics. (DOCX)

S2 Table Analysis on HBsAg titer over time (run-in period). (DOCX)

S3 Table Analysis on log(HBsAg) titer over time (run-in period). (DOCX)

S4 Table Analysis on CKD over time (run-in period). (DOCX)

S5 Table Analysis on HBsAg titer over time (paralled period). (DOCX)

S6 Table Analysis on log(HBsAg) titer over time (paralled period). (DOCX)

S7 Table Analysis on CKD over time (paralled period). (DOCX)

S8 Table The baseline characteristics of initial telbivudine treated patients receiving rescue therapy by early switch according to roadmap rule (n = 21) or by switch on the time of drug resistance (n = 54). (DOCX)
