# Supplementary material for: Kinetics of hepatitis B surface antigen and estimated glomerular filtration rate in telbivudine-treated hepatitis B patients with different rescue strategies
Source: PLoS One. 2020 Aug 12;15(8):e0237586. doi: 10.1371/journal.pone.0237586 (PMC7423127; doi:10.1371/journal.pone.0237586)
Supplement: S1 Table — (DOCX) [file pone.0237586.s001.docx]

##### S1 Table: Summary of Demographics

______________________________________________________________________________

Add-on Adefovir Switch to Tenofovir

Variable N=58 N=44 p-value

______________________________________________________________________________

Sex (column 74) 0.2318

Male 42 ( 72.4%) 37 ( 84.1%)

Female 16 ( 27.6%) 7 ( 15.9%)

Age (year) (column 73) 0.0143

N 58 44

Mean (SD) 47.1 ( 12.7) 53.4 ( 12.6)

Median 47.5 53.5

(Min., Max.) ( 20.0, 80.0) ( 30.0, 87.0)

Roadmap (column 3) 0.8267

Yes 40 ( 69.0%) 32 ( 72.7%)

No 18 ( 31.0%) 12 ( 27.3%)

Reason (column 5) <.0001

Viral breakthrough YMDD (+) 25 ( 43.1%) 12 ( 27.3%)

Viral breakthrough YMDD (-) 14 ( 24.1%) 3 ( 6.8%)

Myopathy 0 ( 0.0%) 27 ( 61.4%)

Early add-on 19 ( 32.8%) 2 ( 4.5%)

Diabetes (column 10) 0.2301

Yes 5 ( 8.6%) 8 ( 18.2%)

No 53 ( 91.4%) 36 ( 81.8%)

Alcohol abuse (> 60 g/day, > 5 year) (column 11) 0.4589

Yes 10 ( 17.2%) 11 ( 25.0%)

No 48 ( 82.8%) 33 ( 75.0%)

Smoking (column 12) 0.3657

Yes 13 ( 22.4%) 14 ( 31.8%)

No 45 ( 77.6%) 30 ( 68.2%)

BMI (kg/m^2) (column 13) 0.8228

N 58 44

Mean (SD) 24.3 ( 3.1) 24.1 ( 4.0)

Median 24.5 24.0

(Min., Max.) ( 18.0, 32.0) ( 17.0, 37.0)

BMI level (column 14) 0.4261

<= 23 24 ( 41.4%) 22 ( 50.0%)

> 23 34 ( 58.6%) 22 ( 50.0%)

Genotype (column 15) 0.4416

Type B 36 ( 62.1%) 22 ( 50.0%)

Type C 17 ( 29.3%) 17 ( 38.6%)

Unknown 5 ( 8.6%) 5 ( 11.4%)

HBeAg status (column 16) 0.0729

Positive 32 ( 55.2%) 16 ( 36.4%)

Negative 26 ( 44.8%) 28 ( 63.6%)

Duration of Sebivo (month) (column 6) 0.0595

N 58 44

Mean (SD) 17.5 ( 15.4) 22.8 ( 11.9)

Median 12.0 19.5

(Min., Max.) ( 6.0, 73.0) ( 6.0, 55.0)

Duration of add-on/switch (month) (column 7&8) 0.0003

N 58 44

Mean (SD) 45.3 ( 17.9) 33.9 ( 10.7)

Median 40.5 33.0

(Min., Max.) ( 11.0, 84.0) ( 12.0, 56.0)

HBV DNA at baseline (IU/ml) (column 17) 0.0042

N 58 44

Mean (SD) 250167332 (517609783) 20584404 ( 53859742)

Median 5003553 954232

(Min., Max.) (350, 2448436747) (106, 284503296)

Log(HBV DNA) at baseline (column 18) 0.0118

N 58 44

Mean (SD) 6.65 ( 1.88) 5.75 ( 1.54)

Median 6.69 5.98

(Min., Max.) ( 2.54, 9.39) ( 2.03, 8.45)

Albumin at baseline (g/dl) (column 19) 0.0088

N 58 44

Mean (SD) 4.3 ( 0.4) 4.0 ( 0.6)

Median 4.3 4.2

(Min., Max.) ( 2.7, 4.8) ( 2.6, 4.7)

GPT at baseline (U/L) (column 22) 0.0565

N 58 44

Mean (SD) 222.2 ( 255.6) 136.7 ( 166.3)

Median 127.0 80.5

(Min., Max.) ( 31.0, 1207.0) ( 13.0, 979.0)

YMDD before change treat (column 40) 0.0886

Yes 23 ( 39.7%) 10 ( 22.7%)

No 35 ( 60.3%) 34 ( 77.3%)

HBsAg at baseline (IU/ml) (column 78) 0.0427

N 58 44

Mean (SD) 54274 (157901) 5085 ( 18587)

Median 2081 861

(Min., Max.) (28, 823801) ( 8, 118901)

Log(HBsAg) at baseline (column 79) 0.0010

N 58 44

Mean (SD) 3.50 ( 1.08) 2.83 ( 0.85)

Median 3.32 2.93

(Min., Max.) ( 1.44, 5.92) ( 0.91, 5.08)

CKD at baseline (%) (column 108) 0.0161

N 58 44

Mean (SD) 86.2 ( 15.9) 78.0 ( 17.9)

Median 87.7 78.8

(Min., Max.) ( 39.7, 122.5) ( 35.7, 119.0)

______________________________________________________________________________

p-value: Group comparison using t test per one-way ANOVA

Fisher's exact test for unordered categorical data
