# Supplementary material for: Kinetics of hepatitis B surface antigen and estimated glomerular filtration rate in telbivudine-treated hepatitis B patients with different rescue strategies
Source: PLoS One. 2020 Aug 12;15(8):e0237586. doi: 10.1371/journal.pone.0237586 (PMC7423127; doi:10.1371/journal.pone.0237586)
Supplement: S2 Table — (DOCX) [file pone.0237586.s002.docx]

##### S2 Table: Analysis on HBsAg Titer Over Time (Run-in Period)

______________________________________________________________________________

Add-on Adefovir Switch to Tenofovir

HBsAg (IU/ml) N=58 N=44 p-value

______________________________________________________________________________

Baseline

N 58 44

Mean (SD) 54274 (157901) 5085 ( 18587) 0.0427

Median 2081 861

(Min., Max.) ( 28, 823801) ( 8, 118901)

Month 3

N 58 41

Mean (SD) 6723 ( 18455) 2302 ( 6367) 0.1445

Median 1294 529

(Min., Max.) ( 38, 90300) ( 12, 39163)

Mean Change from Baseline

Mean (SD) -47551 (152347) -2942 ( 18544) 0.4149

Median -47 -4

(Min., Max.) (-823339, 1714) (-118124, 7138)

intra p-value 0.0208 0.3158

Adjust Group Difference (LsMean with 95% CI) 2367 ( -3370, 8103)

Month 6

N 58 44

Mean (SD) 9235 ( 39683) 3757 ( 15322) 0.3879

Median 1284 585

(Min., Max.) ( 52, 260200) ( 10, 101659)

Mean Change from Baseline

Mean (SD) -45039 (156398) -1328 ( 20402) 0.6005

Median -94 0

(Min., Max.) (-823067, 87599) (-118267, 62496)

intra p-value 0.0324 0.6680

Adjust Group Difference (LsMean with 95% CI) 3359 ( -9329, 16048)

Month 12

N 32 34

Mean (SD) 2530 ( 3295) 1249 ( 2252) 0.0685

Median 1682 567

(Min., Max.) ( 41, 16319) ( 5, 11247)

Mean Change from Baseline

Mean (SD) -48479 (157384) -3697 ( 20331) 0.1488

Median 28 -6

(Min., Max.) (-822245, 4601) (-118276, 5640)

intra p-value 0.0913 0.2967

Adjust Group Difference (LsMean with 95% CI) 1012 ( -372, 2396)

Month 18

N 21 27

Mean (SD) 12709 ( 50099) 1356 ( 2225) 0.2442

Median 1155 623

(Min., Max.) ( 62, 231245) ( 22, 9334)

Mean Change from Baseline

Mean (SD) 9286 ( 50739) -4375 ( 22818) 0.2523

Median 86 21

(Min., Max.) ( -23222, 228979) (-118147, 7225)

intra p-value 0.4115 0.3283

Adjust Group Difference (LsMean with 95% CI) 11308 ( -8332, 30949)

Month 24

N 14 19

Mean (SD) 1289 ( 1226) 1420 ( 2171) 0.8399

Median 993 883

(Min., Max.) ( 70, 4754) ( 11, 9302)

Mean Change from Baseline

Mean (SD) -2726 ( 7607) -6073 ( 27236) 0.8193

Median -22 -13

(Min., Max.) ( -23066, 2015) (-118155, 7193)

intra p-value 0.2030 0.3440

Adjust Group Difference (LsMean with 95% CI) -152 ( -1495, 1192)

Month 30

N 13 13

Mean (SD) 920 ( 893) 1884 ( 3535) 0.3501

Median 625 742

(Min., Max.) ( 62, 3477) ( 148, 13396)

Mean Change from Baseline

Mean (SD) -3305 ( 7668) -9327 ( 32790) 0.3530

Median -342 -155

(Min., Max.) ( -23010, 738) (-118256, 3847)

intra p-value 0.1462 0.3253

Adjust Group Difference (LsMean with 95% CI) -990 ( -3151, 1170)

Month 36

N 6 7

Mean (SD) 575 ( 251) 1673 ( 1774) 0.1633

Median 646 829

(Min., Max.) ( 169, 810) ( 140, 5182)

Mean Change from Baseline

Mean (SD) -3320 ( 7098) -476 ( 2558) 0.2083

Median -594 -264

(Min., Max.) ( -17762, 291) ( -5757, 2133)

intra p-value 0.3037 0.6397

Adjust Group Difference (LsMean with 95% CI) -1051 ( -2793, 690)

______________________________________________________________________________

p-value: Group comparison using t test per one-way ANCOVA w/i or w/o covariate
