# Supplementary material for: Kinetics of hepatitis B surface antigen and estimated glomerular filtration rate in telbivudine-treated hepatitis B patients with different rescue strategies
Source: PLoS One. 2020 Aug 12;15(8):e0237586. doi: 10.1371/journal.pone.0237586 (PMC7423127; doi:10.1371/journal.pone.0237586)
Supplement: S3 Table — (DOCX) [file pone.0237586.s003.docx]

##### S3 Table: Analysis on log(HBsAg) Titer Over Time (Run-in Period)

______________________________________________________________________________

Add-on Adefovir Switch to Tenofovir

log(HBsAg) N=58 N=44 p-value

______________________________________________________________________________

Baseline

N 58 44

Mean (SD) 3.50 ( 1.08) 2.83 ( 0.85) 0.0010

Median 3.32 2.93

(Min., Max.) ( 1.44, 5.92) ( 0.91, 5.08)

Month 3

N 58 41

Mean (SD) 3.14 ( 0.71) 2.59 ( 0.86) 0.0007

Median 3.11 2.72

(Min., Max.) ( 1.58, 4.96) ( 1.08, 4.59)

Mean Change from Baseline

Mean (SD) -0.35 ( 0.80) -0.21 ( 0.62) 0.1461

Median -0.03 -0.08

(Min., Max.) ( -3.25, 1.80) ( -2.18, 0.98)

intra p-value 0.0013 0.0373

Adjust Group Difference (LsMean with 95% CI) 0.18 ( -0.06, 0.42)

Month 6

N 58 44

Mean (SD) 3.08 ( 0.67) 2.74 ( 0.77) 0.0178

Median 3.11 2.77

(Min., Max.) ( 1.72, 5.42) ( 1.00, 5.01)

Mean Change from Baseline

Mean (SD) -0.41 ( 0.82) -0.09 ( 0.69) 0.7615

Median -0.05 0.00

(Min., Max.) ( -3.05, 0.70) ( -2.27, 1.53)

intra p-value 0.0003 0.3830

Adjust Group Difference (LsMean with 95% CI) 0.04 ( -0.20, 0.27)

Month 12

N 32 34

Mean (SD) 3.11 ( 0.56) 2.69 ( 0.67) 0.0070

Median 3.23 2.75

(Min., Max.) ( 1.62, 4.21) ( 0.69, 4.05)

Mean Change from Baseline

Mean (SD) -0.28 ( 0.88) -0.12 ( 0.85) 0.0887

Median 0.03 -0.01

(Min., Max.) ( -2.72, 0.83) ( -2.28, 1.73)

intra p-value 0.0786 0.4328

Adjust Group Difference (LsMean with 95% CI) 0.25 ( -0.04, 0.54)

Month 18

N 21 27

Mean (SD) 3.14 ( 0.71) 2.74 ( 0.64) 0.0449

Median 3.06 2.79

(Min., Max.) ( 1.79, 5.36) ( 1.34, 3.97)

Mean Change from Baseline

Mean (SD) 0.04 ( 0.75) -0.05 ( 0.82) 0.1196

Median 0.07 0.01

(Min., Max.) ( -1.81, 2.01) ( -2.20, 1.57)

intra p-value 0.8084 0.7619

Adjust Group Difference (LsMean with 95% CI) 0.29 ( -0.08, 0.66)

Month 24

N 14 19

Mean (SD) 2.90 ( 0.50) 2.79 ( 0.66) 0.6107

Median 2.99 2.95

(Min., Max.) ( 1.84, 3.68) ( 1.04, 3.97)

Mean Change from Baseline

Mean (SD) -0.19 ( 0.69) -0.17 ( 0.75) 0.7460

Median 0.00 -0.01

(Min., Max.) ( -2.01, 0.47) ( -2.20, 0.89)

intra p-value 0.3285 0.3448

Adjust Group Difference (LsMean with 95% CI) 0.06 ( -0.33, 0.46)

Month 30

N 13 13

Mean (SD) 2.78 ( 0.45) 2.94 ( 0.49) 0.3894

Median 2.80 2.87

(Min., Max.) ( 1.79, 3.54) ( 2.17, 4.13)

Mean Change from Baseline

Mean (SD) -0.31 ( 0.65) -0.24 ( 0.86) 0.4278

Median -0.20 -0.08

(Min., Max.) ( -2.00, 0.35) ( -2.27, 1.31)

intra p-value 0.1109 0.3326

Adjust Group Difference (LsMean with 95% CI) -0.14 ( -0.50, 0.22)

Month 36

N 6 7

Mean (SD) 2.71 ( 0.26) 2.98 ( 0.53) 0.2734

Median 2.81 2.92

(Min., Max.) ( 2.23, 2.91) ( 2.15, 3.71)

Mean Change from Baseline

Mean (SD) -0.47 ( 0.79) -0.24 ( 0.66) 0.2485

Median -0.25 -0.18

(Min., Max.) ( -2.03, 0.19) ( -1.62, 0.28)

intra p-value 0.2092 0.3684

Adjust Group Difference (LsMean with 95% CI) -0.29 ( -0.82, 0.24)

______________________________________________________________________________

p-value: Group comparison using t test per one-way ANCOVA w/i or w/o covariate
