# Supplementary material for: Kinetics of hepatitis B surface antigen and estimated glomerular filtration rate in telbivudine-treated hepatitis B patients with different rescue strategies
Source: PLoS One. 2020 Aug 12;15(8):e0237586. doi: 10.1371/journal.pone.0237586 (PMC7423127; doi:10.1371/journal.pone.0237586)
Supplement: S4 Table — (DOCX) [file pone.0237586.s004.docx]

##### S4 Table: Analysis on CKD Over Time (Run-in Period)

______________________________________________________________________________

Add-on Adefovir Switch to Tenofovir

CKD (%) N=58 N=44 p-value

______________________________________________________________________________

Baseline

N 58 44

Mean (SD) 86.2 ( 15.9) 78.0 ( 17.9) 0.0161

Median 87.7 78.8

(Min., Max.) ( 39.7, 122.5) ( 35.7, 119.0)

Month 3

N 58 44

Mean (SD) 83.2 ( 15.1) 78.5 ( 18.2) 0.1516

Median 82.4 77.6

(Min., Max.) ( 40.1, 119.1) ( 31.4, 118.4)

Mean Change from Baseline

Mean (SD) -3.0 ( 9.5) 0.5 ( 9.9) 0.3067

Median 0.0 1.0

(Min., Max.) ( -26.1, 22.6) ( -30.5, 21.8)

intra p-value 0.0212 0.7413

Adjust Group Difference (LsMean with 95% CI) -2.0 ( -5.7, 1.8)

Month 6

N 58 44

Mean (SD) 83.8 ( 14.4) 76.9 ( 18.8) 0.0388

Median 86.8 75.9

(Min., Max.) ( 39.5, 113.4) ( 32.9, 117.6)

Mean Change from Baseline

Mean (SD) -2.4 ( 9.7) -1.0 ( 13.4) 0.7404

Median -0.4 -2.0

(Min., Max.) ( -26.4, 22.6) ( -48.8, 25.6)

intra p-value 0.0678 0.6106

Adjust Group Difference (LsMean with 95% CI) 0.7 ( -3.6, 5.1)

Month 9

N 39 41

Mean (SD) 80.7 ( 14.5) 80.0 ( 17.9) 0.8430

Median 80.6 82.3

(Min., Max.) ( 39.9, 112.6) ( 39.4, 119.4)

Mean Change from Baseline

Mean (SD) -4.4 ( 11.9) 2.1 ( 10.6) 0.0568

Median -5.4 0.8

(Min., Max.) ( -32.3, 33.6) ( -27.6, 21.1)

intra p-value 0.0263 0.2164

Adjust Group Difference (LsMean with 95% CI) -4.6 ( -9.3, 0.1)

Month 12

N 32 37

Mean (SD) 82.6 ( 16.2) 86.6 ( 20.0) 0.3748

Median 85.2 84.2

(Min., Max.) ( 36.7, 107.2) ( 36.8, 118.2)

Mean Change from Baseline

Mean (SD) -1.2 ( 11.9) 8.3 ( 16.1) 0.0193

Median -0.7 7.8

(Min., Max.) ( -37.4, 24.7) ( -32.9, 48.5)

intra p-value 0.5731 0.0033

Adjust Group Difference (LsMean with 95% CI) -8.0 ( -14.7, -1.3)

Month 15

N 26 32

Mean (SD) 84.5 ( 15.0) 86.1 ( 16.7) 0.6958

Median 84.6 85.7

(Min., Max.) ( 40.6, 108.7) ( 38.3, 121.4)

Mean Change from Baseline

Mean (SD) 0.2 ( 12.4) 7.3 ( 13.4) 0.1035

Median -0.7 8.0

(Min., Max.) ( -23.5, 24.9) ( -24.5, 34.3)

intra p-value 0.9375 0.0043

Adjust Group Difference (LsMean with 95% CI) -5.4 ( -11.8, 1.1)

Month 18

N 21 28

Mean (SD) 81.8 ( 14.4) 86.2 ( 18.1) 0.3725

Median 80.8 89.2

(Min., Max.) ( 52.4, 108.5) ( 34.8, 119.2)

Mean Change from Baseline

Mean (SD) -1.5 ( 11.3) 6.7 ( 14.5) 0.0578

Median -1.3 8.6

(Min., Max.) ( -20.1, 18.3) ( -21.4, 35.4)

intra p-value 0.5576 0.0214

Adjust Group Difference (LsMean with 95% CI) -7.0 ( -14.2, 0.2)

Month 21

N 15 20

Mean (SD) 81.4 ( 14.3) 89.2 ( 16.9) 0.1597

Median 78.7 90.7

(Min., Max.) ( 56.3, 110.3) ( 50.3, 123.3)

Mean Change from Baseline

Mean (SD) -2.6 ( 11.6) 9.3 ( 14.2) 0.0162

Median -1.1 4.4

(Min., Max.) ( -28.2, 17.1) ( -15.6, 37.4)

intra p-value 0.4019 0.0083

Adjust Group Difference (LsMean with 95% CI) -10.5 ( -18.8, -2.1)

Month 24

N 14 19

Mean (SD) 85.1 ( 11.7) 89.5 ( 12.9) 0.3293

Median 83.6 91.8

(Min., Max.) ( 70.4, 112.0) ( 67.6, 118.6)

Mean Change from Baseline

Mean (SD) -0.4 ( 13.2) 10.1 ( 15.3) 0.1019

Median -0.6 11.0

(Min., Max.) ( -20.4, 24.1) ( -12.4, 41.0)

intra p-value 0.9030 0.0100

Adjust Group Difference (LsMean with 95% CI) -6.7 ( -14.8, 1.4)

Month 27

N 14 15

Mean (SD) 87.6 ( 13.6) 85.2 ( 14.8) 0.6607

Median 86.3 82.4

(Min., Max.) ( 67.0, 113.5) ( 62.6, 115.1)

Mean Change from Baseline

Mean (SD) 2.0 ( 12.1) 11.3 ( 20.1) 0.8921

Median 5.8 16.2

(Min., Max.) ( -23.5, 14.5) ( -17.3, 49.2)

intra p-value 0.5481 0.0477

Adjust Group Difference (LsMean with 95% CI) -0.8 ( -12.6, 11.0)

Month 30

N 13 13

Mean (SD) 85.2 ( 15.0) 94.2 ( 13.6) 0.1219

Median 85.7 95.8

(Min., Max.) ( 64.3, 113.9) ( 64.9, 122.4)

Mean Change from Baseline

Mean (SD) 0.0 ( 12.4) 15.8 ( 14.6) 0.0124

Median -4.7 12.0

(Min., Max.) ( -16.3, 24.7) ( -1.7, 41.3)

intra p-value 0.9953 0.0021

Adjust Group Difference (LsMean with 95% CI) -12.7 ( -22.4, -3.0)

Month 33

N 7 9

Mean (SD) 84.6 ( 11.8) 85.1 ( 10.8) 0.9376

Median 81.4 82.2

(Min., Max.) ( 72.7, 99.9) ( 69.7, 99.0)

Mean Change from Baseline

Mean (SD) -1.1 ( 7.3) 11.4 ( 11.1) 0.1199

Median -2.8 6.6

(Min., Max.) ( -9.2, 11.9) ( -1.3, 33.0)

intra p-value 0.7143 0.0149

Adjust Group Difference (LsMean with 95% CI) -7.5 ( -17.3, 2.2)

Month 36

N 6 7

Mean (SD) 81.7 ( 14.2) 91.0 ( 11.4) 0.2125

Median 81.0 96.4

(Min., Max.) ( 64.3, 99.9) ( 69.3, 101.0)

Mean Change from Baseline

Mean (SD) -6.8 ( 10.8) 14.8 ( 9.3) 0.0161

Median -8.6 13.2

(Min., Max.) ( -17.5, 11.9) ( 3.5, 28.1)

intra p-value 0.1797 0.0056

Adjust Group Difference (LsMean with 95% CI) -18.3 ( -32.3, -4.2)

______________________________________________________________________________

p-value: Group comparison using t test per one-way ANCOVA w/i or w/o covariate
