# Supplementary material for: Kinetics of hepatitis B surface antigen and estimated glomerular filtration rate in telbivudine-treated hepatitis B patients with different rescue strategies
Source: PLoS One. 2020 Aug 12;15(8):e0237586. doi: 10.1371/journal.pone.0237586 (PMC7423127; doi:10.1371/journal.pone.0237586)
Supplement: S5 Table — (DOCX) [file pone.0237586.s005.docx]

##### S5 Table: Analysis on HBsAg Titer Over Time (Paralled Period)

______________________________________________________________________________

Add-on Adefovir Switch to Tenofovir

HBsAg (IU/ml) N=58 N=44 p-value

______________________________________________________________________________

Baseline

N 58 44

Mean (SD) 9718 ( 39651) 3723 ( 15361) 0.3447

Median 1223 491

(Min., Max.) ( 52, 260200) ( 10, 101659)

Month 3

N 58 43

Mean (SD) 5162 ( 22066) 1307 ( 2715) 0.2580

Median 1259 622

(Min., Max.) ( 37, 168690) ( 28, 15163)

Mean Change from Baseline (Par

Mean (SD) -4556 ( 34142) -2497 ( 13243) 0.4454

Median -31 4

(Min., Max.) (-257793, 15932) ( -86496, 2159)

intra p-value 0.3138 0.2232

Adjust Group Difference (LsMean with 95% CI) 2254 ( -3583, 8090)

Month 6

N 57 43

Mean (SD) 8488 ( 43805) 6536 ( 36528) 0.8134

Median 1153 652

(Min., Max.) ( 34, 328777) ( 23, 240300)

Mean Change from Baseline (Par

Mean (SD) -1399 ( 41907) 2731 ( 21314) 0.7161

Median -28 22

(Min., Max.) (-257977, 168177) ( -9183, 138641)

intra p-value 0.8019 0.4055

Adjust Group Difference (LsMean with 95% CI) -2495 (-16070, 11080)

Month 12

N 57 44

Mean (SD) 3444 ( 9133) 1411 ( 3449) 0.1645

Median 1341 551

(Min., Max.) ( 9, 63700) ( 18, 20565)

Mean Change from Baseline (Par

Mean (SD) -6440 ( 36588) -2312 ( 12295) 0.2998

Median -113 -60

(Min., Max.) (-258195, 25472) ( -81094, 2444)

intra p-value 0.1893 0.2191

Adjust Group Difference (LsMean with 95% CI) 1311 ( -1184, 3806)

Month 18

N 55 42

Mean (SD) 2498 ( 5247) 2468 ( 8761) 0.9834

Median 947 419

(Min., Max.) ( 29, 34530) ( 9, 53526)

Mean Change from Baseline (Par

Mean (SD) -7661 ( 39548) -1388 ( 8313) 0.7050

Median -140 -83

(Min., Max.) (-255200, 31942) ( -48133, 19801)

intra p-value 0.1566 0.2856

Adjust Group Difference (LsMean with 95% CI) -505 ( -3144, 2134)

Month 24

N 53 38

Mean (SD) 1771 ( 2880) 1409 ( 4389) 0.6362

Median 748 328

(Min., Max.) ( 12, 17542) ( 8, 25000)

Mean Change from Baseline (Par

Mean (SD) -5146 ( 35621) -2488 ( 12699) 0.7231

Median -80 -139

(Min., Max.) (-258603, 14954) ( -76659, 11511)

intra p-value 0.2978 0.2349

Adjust Group Difference (LsMean with 95% CI) 263 ( -1207, 1733)

Month 30

N 45 30

Mean (SD) 2505 ( 6516) 1470 ( 4356) 0.4479

Median 893 390

(Min., Max.) ( 6, 43176) ( 7, 23863)

Mean Change from Baseline (Par

Mean (SD) -5967 ( 39443) -3333 ( 14292) 0.4893

Median -280 -245

(Min., Max.) (-259060, 41389) ( -77796, 4106)

intra p-value 0.3157 0.2116

Adjust Group Difference (LsMean with 95% CI) 941 ( -1758, 3640)

Month 36

N 40 20

Mean (SD) 1649 ( 2603) 412 ( 426) 0.0399

Median 817 277

(Min., Max.) ( 2, 14409) ( 8, 1668)

Mean Change from Baseline (Par

Mean (SD) -7753 ( 41175) -704 ( 1952) 0.0412

Median -273 -223

(Min., Max.) (-259219, 11821) ( -8465, 1539)

intra p-value 0.2409 0.1235

Adjust Group Difference (LsMean with 95% CI) 1248 ( 52, 2444)

Month 42

N 25 11

Mean (SD) 1937 ( 2660) 419 ( 366) 0.0702

Median 781 423

(Min., Max.) ( 36, 9284) ( 5, 1088)

Mean Change from Baseline (Par

Mean (SD) -10694 ( 51884) -541 ( 666) 0.0680

Median -343 -441

(Min., Max.) (-259512, 6696) ( -1896, 483)

intra p-value 0.3130 0.0225

Adjust Group Difference (LsMean with 95% CI) 1563 ( -122, 3249)

Month 48

N 20 5

Mean (SD) 1582 ( 2307) 267 ( 244) 0.2227

Median 342 337

(Min., Max.) ( 5, 7912) ( 13, 580)

Mean Change from Baseline (Par

Mean (SD) -14129 ( 57942) -644 ( 919) 0.2137

Median -909 -407

(Min., Max.) (-259765, 5736) ( -1964, 294)

intra p-value 0.2891 0.1922

Adjust Group Difference (LsMean with 95% CI) 1376 ( -853, 3606)

Month 54

N 20 1

Mean (SD) 1174 ( 1457) 5 ( ) 0.4434

Median 491 5

(Min., Max.) ( 13, 5348) ( 5, 5)

Mean Change from Baseline (Par

Mean (SD) -14322 ( 57874) -415 ( ) 0.4399

Median -310 -415

(Min., Max.) (-259778, 2760) ( -415, -415)

intra p-value 0.2822

Adjust Group Difference (LsMean with 95% CI) 1207 ( -2004, 4418)

______________________________________________________________________________

p-value: Group comparison using t test per one-way ANCOVA w/i or w/o covariate
