# Supplementary material for: Kinetics of hepatitis B surface antigen and estimated glomerular filtration rate in telbivudine-treated hepatitis B patients with different rescue strategies
Source: PLoS One. 2020 Aug 12;15(8):e0237586. doi: 10.1371/journal.pone.0237586 (PMC7423127; doi:10.1371/journal.pone.0237586)
Supplement: S6 Table — (DOCX) [file pone.0237586.s006.docx]

##### S6 Table: Analysis on log(HBsAg) Titer Over Time (Paralled Period)

______________________________________________________________________________

Add-on Adefovir Switch to Tenofovir

log(HBsAg) N=58 N=44 p-value

______________________________________________________________________________

Baseline

N 58 44

Mean (SD) 3.12 ( 0.71) 2.73 ( 0.74) 0.0095

Median 3.09 2.69

(Min., Max.) ( 1.72, 5.42) ( 1.00, 5.01)

Month 3

N 58 43

Mean (SD) 3.06 ( 0.67) 2.74 ( 0.54) 0.0139

Median 3.10 2.79

(Min., Max.) ( 1.57, 5.23) ( 1.45, 4.18)

Mean Change from Baseline (Par

Mean (SD) -0.06 ( 0.45) 0.00 ( 0.48) 0.4287

Median -0.03 0.01

(Min., Max.) ( -2.03, 1.13) ( -0.95, 1.55)

intra p-value 0.3245 0.9738

Adjust Group Difference (LsMean with 95% CI) 0.06 ( -0.10, 0.23)

Month 6

N 57 43

Mean (SD) 3.08 ( 0.67) 2.77 ( 0.67) 0.0266

Median 3.06 2.81

(Min., Max.) ( 1.53, 5.52) ( 1.36, 5.38)

Mean Change from Baseline (Par

Mean (SD) -0.06 ( 0.39) 0.03 ( 0.54) 0.8278

Median -0.02 0.05

(Min., Max.) ( -2.07, 1.33) ( -1.33, 1.66)

intra p-value 0.2500 0.7272

Adjust Group Difference (LsMean with 95% CI) 0.02 ( -0.16, 0.19)

Month 12

N 57 44

Mean (SD) 2.99 ( 0.72) 2.62 ( 0.66) 0.0080

Median 3.13 2.74

(Min., Max.) ( 0.96, 4.80) ( 1.25, 4.31)

Mean Change from Baseline (Par

Mean (SD) -0.14 ( 0.42) -0.12 ( 0.67) 0.2885

Median -0.10 -0.07

(Min., Max.) ( -2.11, 1.04) ( -2.22, 1.89)

intra p-value 0.0179 0.2523

Adjust Group Difference (LsMean with 95% CI) 0.11 ( -0.09, 0.31)

Month 18

N 55 42

Mean (SD) 2.92 ( 0.69) 2.56 ( 0.75) 0.0170

Median 2.98 2.62

(Min., Max.) ( 1.47, 4.54) ( 0.94, 4.73)

Mean Change from Baseline (Par

Mean (SD) -0.19 ( 0.43) -0.16 ( 0.77) 0.3501

Median -0.15 -0.10

(Min., Max.) ( -1.72, 1.13) ( -2.62, 1.52)

intra p-value 0.0017 0.1936

Adjust Group Difference (LsMean with 95% CI) 0.11 ( -0.12, 0.34)

Month 24

N 53 38

Mean (SD) 2.82 ( 0.69) 2.40 ( 0.80) 0.0099

Median 2.87 2.52

(Min., Max.) ( 1.07, 4.24) ( 0.92, 4.40)

Mean Change from Baseline (Par

Mean (SD) -0.23 ( 0.48) -0.32 ( 0.78) 0.1179

Median -0.09 -0.32

(Min., Max.) ( -2.21, 0.83) ( -2.65, 1.29)

intra p-value 0.0010 0.0154

Adjust Group Difference (LsMean with 95% CI) 0.20 ( -0.05, 0.45)

Month 30

N 45 30

Mean (SD) 2.87 ( 0.72) 2.48 ( 0.79) 0.0308

Median 2.95 2.59

(Min., Max.) ( 0.76, 4.64) ( 0.85, 4.38)

Mean Change from Baseline (Par

Mean (SD) -0.24 ( 0.58) -0.32 ( 0.78) 0.1793

Median -0.16 -0.25

(Min., Max.) ( -2.36, 1.38) ( -2.60, 1.37)

intra p-value 0.0076 0.0348

Adjust Group Difference (LsMean with 95% CI) 0.20 ( -0.09, 0.49)

Month 36

N 40 20

Mean (SD) 2.76 ( 0.78) 2.31 ( 0.62) 0.0274

Median 2.91 2.44

(Min., Max.) ( 0.21, 4.16) ( 0.90, 3.22)

Mean Change from Baseline (Par

Mean (SD) -0.35 ( 0.59) -0.29 ( 0.68) 0.4232

Median -0.26 -0.35

(Min., Max.) ( -2.42, 0.75) ( -1.72, 1.11)

intra p-value 0.0007 0.0688

Adjust Group Difference (LsMean with 95% CI) 0.13 ( -0.20, 0.46)

Month 42

N 25 11

Mean (SD) 2.85 ( 0.70) 2.28 ( 0.76) 0.0346

Median 2.89 2.63

(Min., Max.) ( 1.56, 3.97) ( 0.66, 3.04)

Mean Change from Baseline (Par

Mean (SD) -0.36 ( 0.60) -0.46 ( 0.76) 0.1944

Median -0.25 -0.37

(Min., Max.) ( -2.58, 0.55) ( -1.97, 0.73)

intra p-value 0.0070 0.0712

Adjust Group Difference (LsMean with 95% CI) 0.30 ( -0.16, 0.75)

Month 48

N 20 5

Mean (SD) 2.61 ( 0.88) 2.08 ( 0.76) 0.2305

Median 2.52 2.53

(Min., Max.) ( 0.71, 3.90) ( 1.12, 2.76)

Mean Change from Baseline (Par

Mean (SD) -0.66 ( 0.83) -0.45 ( 0.80) 0.7702

Median -0.52 -0.65

(Min., Max.) ( -2.78, 0.67) ( -1.50, 0.40)

intra p-value 0.0022 0.2810

Adjust Group Difference (LsMean with 95% CI) 0.12 ( -0.73, 0.97)

Month 54

N 20 1

Mean (SD) 2.66 ( 0.72) 0.73 ( ) 0.0167

Median 2.69 0.73

(Min., Max.) ( 1.11, 3.73) ( 0.73, 0.73)

Mean Change from Baseline (Par

Mean (SD) -0.56 ( 0.74) -1.89 ( ) 0.0240

Median -0.38 -1.89

(Min., Max.) ( -2.79, 0.35) ( -1.89, -1.89)

intra p-value 0.0030

Adjust Group Difference (LsMean with 95% CI) 1.65 ( 0.24, 3.05)

______________________________________________________________________________

p-value: Group comparison using t test per one-way ANCOVA w/i or w/o covariate
