# Supplementary material for: Kinetics of hepatitis B surface antigen and estimated glomerular filtration rate in telbivudine-treated hepatitis B patients with different rescue strategies
Source: PLoS One. 2020 Aug 12;15(8):e0237586. doi: 10.1371/journal.pone.0237586 (PMC7423127; doi:10.1371/journal.pone.0237586)
Supplement: S7 Table — (DOCX) [file pone.0237586.s007.docx]

##### S7 Table: Analysis on CKD Over Time (Paralled Period)

______________________________________________________________________________

Add-on Adefovir Switch to Tenofovir

CKD (%) N=58 N=44 p-value

______________________________________________________________________________

Baseline

N 58 44

Mean (SD) 84.9 ( 15.4) 87.6 ( 20.6) 0.4442

Median 84.7 89.7

(Min., Max.) ( 39.9, 113.9) ( 34.8, 122.4)

Month 3

N 58 44

Mean (SD) 87.6 ( 15.4) 86.3 ( 19.2) 0.7041

Median 87.6 86.6

(Min., Max.) ( 36.9, 118.3) ( 27.3, 117.0)

Mean Change from Baseline (Par

Mean (SD) 2.7 ( 11.6) -0.9 ( 12.8) 0.1756

Median 0.8 -3.0

(Min., Max.) ( -24.0, 31.6) ( -28.0, 37.8)

intra p-value 0.0782 0.6215

Adjust Group Difference (LsMean with 95% CI) 3.0 ( -1.4, 7.5)

Month 6

N 58 42

Mean (SD) 90.3 ( 16.0) 83.5 ( 19.3) 0.0570

Median 90.8 82.5

(Min., Max.) ( 35.9, 118.3) ( 28.5, 119.7)

Mean Change from Baseline (Par

Mean (SD) 5.4 ( 10.8) -3.2 ( 11.8) 0.0002

Median 4.9 -2.7

(Min., Max.) ( -21.0, 30.5) ( -26.2, 31.1)

intra p-value 0.0003 0.0825

Adjust Group Difference (LsMean with 95% CI) 8.2 ( 4.0, 12.4)

Month 9

N 58 43

Mean (SD) 89.1 ( 14.7) 81.8 ( 18.0) 0.0262

Median 91.6 83.2

(Min., Max.) ( 36.5, 120.7) ( 34.5, 119.7)

Mean Change from Baseline (Par

Mean (SD) 4.2 ( 11.6) -5.4 ( 12.0) <.0001

Median 2.3 -5.1

(Min., Max.) ( -15.1, 27.7) ( -32.8, 26.7)

intra p-value 0.0079 0.0047

Adjust Group Difference (LsMean with 95% CI) 8.9 ( 4.7, 13.0)

Month 12

N 56 44

Mean (SD) 90.9 ( 16.2) 78.9 ( 17.2) 0.0005

Median 92.8 79.0

(Min., Max.) ( 40.5, 121.7) ( 32.3, 113.4)

Mean Change from Baseline (Par

Mean (SD) 5.6 ( 12.1) -8.4 ( 10.3) <.0001

Median 4.1 -9.0

(Min., Max.) ( -19.9, 47.5) ( -35.1, 14.7)

intra p-value 0.0010 <.0001

Adjust Group Difference (LsMean with 95% CI) 13.5 ( 9.4, 17.6)

Month 15

N 55 40

Mean (SD) 88.7 ( 18.5) 78.5 ( 18.7) 0.0101

Median 89.7 79.3

(Min., Max.) ( 12.5, 122.2) ( 29.6, 115.8)

Mean Change from Baseline (Par

Mean (SD) 3.9 ( 13.1) -8.4 ( 12.0) <.0001

Median 2.8 -9.8

(Min., Max.) ( -28.5, 34.6) ( -27.4, 24.2)

intra p-value 0.0300 <.0001

Adjust Group Difference (LsMean with 95% CI) 11.9 ( 6.9, 16.9)

Month 18

N 55 41

Mean (SD) 88.4 ( 19.5) 78.8 ( 16.8) 0.0134

Median 86.5 81.8

(Min., Max.) ( 9.1, 118.3) ( 36.8, 113.2)

Mean Change from Baseline (Par

Mean (SD) 3.6 ( 12.3) -7.5 ( 11.4) <.0001

Median 4.1 -8.4

(Min., Max.) ( -30.9, 45.8) ( -26.4, 21.4)

intra p-value 0.0336 0.0001

Adjust Group Difference (LsMean with 95% CI) 10.8 ( 6.1, 15.5)

Month 21

N 54 38

Mean (SD) 86.7 ( 19.2) 78.5 ( 15.8) 0.0331

Median 89.5 78.8

(Min., Max.) ( 8.3, 121.4) ( 39.0, 113.2)

Mean Change from Baseline (Par

Mean (SD) 2.1 ( 11.9) -10.1 ( 13.2) <.0001

Median 2.9 -10.5

(Min., Max.) ( -31.6, 31.3) ( -35.3, 23.5)

intra p-value 0.2062 <.0001

Adjust Group Difference (LsMean with 95% CI) 11.3 ( 6.3, 16.4)

Month 24

N 55 37

Mean (SD) 87.8 ( 18.9) 76.0 ( 16.7) 0.0026

Median 88.3 76.4

(Min., Max.) ( 9.4, 123.4) ( 26.7, 110.9)

Mean Change from Baseline (Par

Mean (SD) 3.5 ( 13.4) -11.0 ( 13.5) <.0001

Median 4.3 -10.3

(Min., Max.) ( -30.7, 29.0) ( -35.2, 22.8)

intra p-value 0.0557 <.0001

Adjust Group Difference (LsMean with 95% CI) 13.8 ( 8.5, 19.1)

Month 27

N 51 31

Mean (SD) 89.3 ( 19.3) 77.0 ( 16.9) 0.0044

Median 92.3 77.7

(Min., Max.) ( 6.8, 115.1) ( 34.5, 112.7)

Mean Change from Baseline (Par

Mean (SD) 5.3 ( 13.9) -11.6 ( 10.9) <.0001

Median 6.1 -13.5

(Min., Max.) ( -33.1, 40.7) ( -26.3, 13.8)

intra p-value 0.0094 <.0001

Adjust Group Difference (LsMean with 95% CI) 15.9 ( 10.2, 21.6)

Month 30

N 45 30

Mean (SD) 88.5 ( 20.1) 76.0 ( 15.4) 0.0052

Median 91.0 80.9

(Min., Max.) ( 5.8, 118.8) ( 38.2, 101.7)

Mean Change from Baseline (Par

Mean (SD) 4.1 ( 13.1) -11.9 ( 12.6) <.0001

Median 4.3 -11.0

(Min., Max.) ( -34.1, 26.4) ( -36.2, 9.6)

intra p-value 0.0411 <.0001

Adjust Group Difference (LsMean with 95% CI) 15.2 ( 9.3, 21.0)

Month 33

N 43 23

Mean (SD) 86.0 ( 20.5) 77.5 ( 16.2) 0.0870

Median 89.6 78.0

(Min., Max.) ( 7.1, 117.7) ( 37.8, 107.2)

Mean Change from Baseline (Par

Mean (SD) 1.2 ( 13.6) -12.9 ( 11.0) 0.0001

Median 3.3 -16.5

(Min., Max.) ( -32.9, 29.6) ( -26.6, 15.3)

intra p-value 0.5595 <.0001

Adjust Group Difference (LsMean with 95% CI) 13.2 ( 6.7, 19.8)

Month 36

N 40 18

Mean (SD) 87.0 ( 19.4) 79.3 ( 15.5) 0.1468

Median 87.3 78.4

(Min., Max.) ( 6.0, 118.0) ( 53.2, 111.2)

Mean Change from Baseline (Par

Mean (SD) 2.7 ( 12.2) -16.2 ( 9.3) <.0001

Median 0.6 -17.5

(Min., Max.) ( -33.9, 24.8) ( -30.8, -0.7)

intra p-value 0.1665 <.0001

Adjust Group Difference (LsMean with 95% CI) 17.8 ( 11.0, 24.6)

Month 39

N 35 14

Mean (SD) 87.3 ( 19.7) 81.2 ( 14.2) 0.3000

Median 88.3 82.9

(Min., Max.) ( 5.9, 119.2) ( 53.2, 110.6)

Mean Change from Baseline (Par

Mean (SD) 3.1 ( 13.1) -16.5 ( 8.5) <.0001

Median 1.2 -17.6

(Min., Max.) ( -34.0, 25.5) ( -26.4, -1.7)

intra p-value 0.1768 <.0001

Adjust Group Difference (LsMean with 95% CI) 17.8 ( 9.7, 26.0)

Month 42

N 29 12

Mean (SD) 87.1 ( 20.7) 84.9 ( 15.8) 0.7443

Median 93.3 85.0

(Min., Max.) ( 6.2, 109.4) ( 52.5, 106.1)

Mean Change from Baseline (Par

Mean (SD) 2.6 ( 13.9) -14.9 ( 11.5) 0.0032

Median 1.8 -12.4

(Min., Max.) ( -33.7, 33.2) ( -34.7, 2.4)

intra p-value 0.3171 0.0009

Adjust Group Difference (LsMean with 95% CI) 15.7 ( 5.6, 25.8)

Month 45

N 23 8

Mean (SD) 87.6 ( 22.4) 86.5 ( 13.2) 0.8970

Median 93.6 90.3

(Min., Max.) ( 6.0, 115.8) ( 61.8, 103.3)

Mean Change from Baseline (Par

Mean (SD) 3.1 ( 13.8) -17.7 ( 8.0) 0.0018

Median 5.1 -19.7

(Min., Max.) ( -33.9, 22.9) ( -25.9, -6.7)

intra p-value 0.2934 0.0004

Adjust Group Difference (LsMean with 95% CI) 20.6 ( 8.3, 32.9)

Month 48

N 21 6

Mean (SD) 84.8 ( 22.8) 87.0 ( 15.4) 0.8303

Median 86.9 91.8

(Min., Max.) ( 6.0, 111.8) ( 57.3, 97.4)

Mean Change from Baseline (Par

Mean (SD) 1.7 ( 15.8) -20.9 ( 8.6) 0.0150

Median 1.7 -20.9

(Min., Max.) ( -33.9, 28.4) ( -31.3, -10.1)

intra p-value 0.6318 0.0019

Adjust Group Difference (LsMean with 95% CI) 21.6 ( 4.6, 38.5)

Month 51

N 18 2

Mean (SD) 85.2 ( 24.4) 85.7 ( 9.9) 0.9784

Median 91.9 85.7

(Min., Max.) ( 9.1, 110.6) ( 78.7, 92.7)

Mean Change from Baseline (Par

Mean (SD) 1.9 ( 13.3) -32.1 ( 9.5) 0.0034

Median 3.5 -32.1

(Min., Max.) ( -30.8, 21.7) ( -38.8, -25.4)

intra p-value 0.5434 0.1313

Adjust Group Difference (LsMean with 95% CI) 39.4 ( 14.9, 63.9)

Month 54

N 18 2

Mean (SD) 85.8 ( 24.1) 91.5 ( 2.8) 0.7491

Median 89.3 91.5

(Min., Max.) ( 5.9, 110.4) ( 89.5, 93.5)

Mean Change from Baseline (Par

Mean (SD) 2.4 ( 16.6) -26.3 ( 2.4) 0.0762

Median 4.2 -26.3

(Min., Max.) ( -34.1, 30.2) ( -28.0, -24.6)

intra p-value 0.5433 0.0417

Adjust Group Difference (LsMean with 95% CI) 27.6 ( -3.2, 58.4)

______________________________________________________________________________

p-value: Group comparison using t test per one-way ANCOVA w/i or w/o covariate
