# Supplementary material for: Kinetics of hepatitis B surface antigen and estimated glomerular filtration rate in telbivudine-treated hepatitis B patients with different rescue strategies
Source: PLoS One. 2020 Aug 12;15(8):e0237586. doi: 10.1371/journal.pone.0237586 (PMC7423127; doi:10.1371/journal.pone.0237586)
Supplement: S8 Table — (DOCX) [file pone.0237586.s008.docx]

S8 table. The baseline characteristics of initial telbivudine treated patients receiving rescue therapy by early switch according to roadmap rule (n=21) or by switch on the time of drug resistance (n=54).

| Characteristics | Early switch by roadmap rule  N=21 | Switch for drug resistance  N=54 | P value |
| --- | --- | --- | --- |
| Age, years [mean (SD)] | 43 (12) | 51 (13) | 0.017^*^ |
| Male/ female | 17/4 | 39/15 | 0.560 |
| Liver cirrhosis, present/ absent | 4/17 | 21/33 | 0.171 |
| HBeAg status, positive/ negative | 15/6 | 23/31 | 0.039* |
| Genotype B / C/ unknown | 12/8/1 | 32/18/4 | 0.874 |
| ALT> 200 U/L, yes/ no | 10/11 | 10/44 | 0.018* |
| HBV DNA > 7 log IU/mL, yes/ no | 14/7 | 15/39 | 0.003^*^ |
| eGFR (CKD-EPI), mL/min/1.73 m^2^  ≥90/89-60/59-30/<30 | 7/14/0/0 | 20/31/2/1 | 0.696 |
| qHBsAg, IU/mL  ≥5000/4999-1000/999-100/<100 | 10/6/5/0 | 11/19/18/6 | 0.072 |
